# Optimizing the contrast agent dose and image acquisition time for renal ultrasound localization microscopy

**DOI:** 10.1186/s41747-026-00850-8

**Published:** 2026-11-13

**Authors:** Jing Zhang, Jiahao Han, Pingping Wang, Lu Chen, Yingchun Wu, Jia Li

**Affiliations:** 1 Department of Ultrasound, Zhongda Hospital, Medical School, Southeast University, Nanjing, China; 2 Ultrasonic Department, The Second Affiliated Hospital of Nanjing Medical University, Nanjing, China

**Keywords:** Kidney, Microbubbles, Perfusion, Sulfur hexafluoride, Ultrasound

## Abstract

**Objective:**

Renal microvascular assessment is essential for kidney function. Ultrasound localization microscopy (ULM) enables super-resolution imaging of renal microvasculature. However, contrast dose and acquisition time need to be optimized. This study explored the effects of contrast agent dose and acquisition time on renal cortical microvascular imaging.

**Materials and methods:**

In this prospective study, healthy volunteers underwent renal contrast-enhanced ultrasound (CEUS) using four sulfur hexafluoride microbubbles doses (0.4, 0.6, 0.8, and 1.2 mL). ULM was performed during cortical (peak enhancement), medullary, and delayed phases. Quantitative CEUS perfusion parameters and ULM-derived microvascular metrics were assessed, and the effects of dose and time were evaluated using repeated-measures ANOVA. The minimal effective dose was predefined using repeated-measures ANOVA with Bonferroni correction as the lowest dose without significant benefit from higher doses.

**Results:**

Twenty-five participants were included. CEUS parameters, including area under the curve, peak intensity, half-time to peak, and acceleration time, demonstrated a clear dose–response relationship, plateauing at doses ≥ 0.6 mL (all *p* ≤ 0.008). ULM imaging was most favorable during the peak enhancement phase. Four distinct patterns of ULM parameters were identified: dose- and time-dependent, dose-dependent, time-dependent, and stable. Among these, the dose- and time-dependent parameters (*e.g*., vessel ratio, maximum velocity, mean velocity) exhibited the most consistent plateau at 0.6 mL, paralleling the CEUS findings.

**Conclusion:**

In healthy, non-obese adults, a 0.6-mL dose at peak enhancement reached a plateau in quantitative performance and may represent a practical minimal effective protocol for renal ULM.

**Key Points:**

***Question*** What are the optimal contrast dose and acquisition time for renal ULM protocols, given that they are not currently standardized?

***Findings*** A minimal effective dose of 0.6 mL at peak enhancement in healthy, non-obese adults was found. Multiparametric ULM shows good to excellent intra- and inter-observer repeatability for protocol optimization.

***Relevance statement*** Renal ULM enables quantitative assessment of cortical microvascular perfusion. This technique provides a reference for optimizing contrast dose and acquisition time, supporting standardized imaging protocols for kidney function evaluation.

**Graphical Abstract:**

**Figure:**
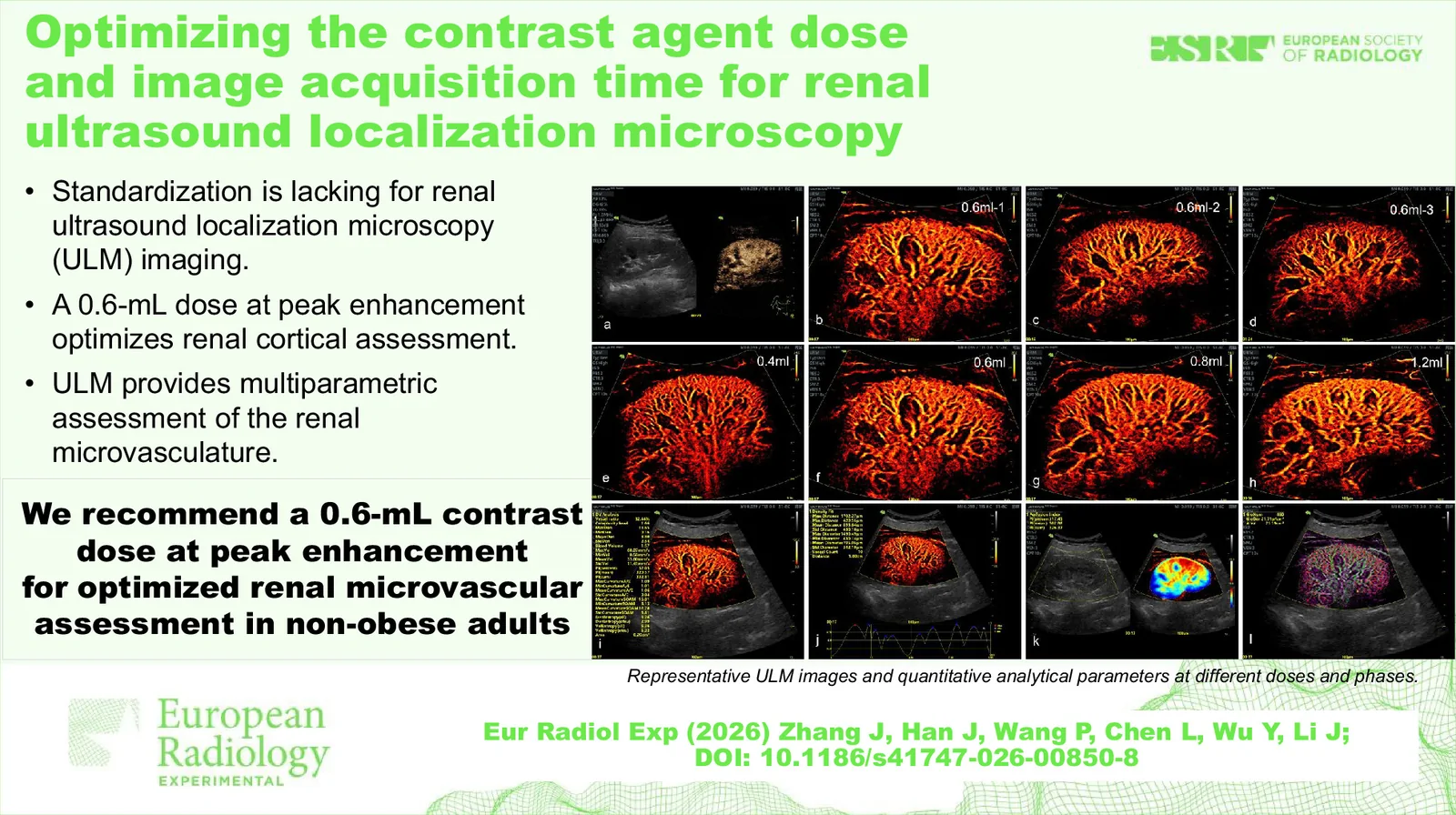

## Background

Evaluation of the renal microvasculature is crucial for understanding kidney function and the pathophysiology of diseases such as ischemia, fibrosis, and transplant rejection [1–5]. However, current clinical imaging techniques, including conventional Doppler ultrasound and contrast-enhanced ultrasound (CEUS), are constrained by acoustic diffraction, limiting spatial resolution to > 100 μm and rendering the cortical microvasculature largely invisible [6]. This resolution gap hinders the early detection of microvascular rarefaction and dysfunction, which are key features of progressive nephropathies.

Ultrasound localization microscopy (ULM) is a super-resolution imaging technique that overcomes the acoustic diffraction limit of conventional ultrasound through the precise localization and tracking of intravascular microbubbles [7–9]. By reconstructing microvascular networks at near-capillary scale, ULM enables visualization of vessels far below the resolution of conventional ultrasound while preserving deep-tissue penetration. Its feasibility has been demonstrated in several organs, highlighting its translational potential for clinical microvascular imaging [10–14]. For renal applications, ULM offers a novel minimally invasive approach to visualize the cortical capillary network *in vivo*.

However, translation of ULM to renal imaging faces unique physiological and technical challenges. The kidney’s high cortical perfusion (90–95% of total flow) creates a dense and complex microvascular environment [15]. This physiology necessitates precise optimization of two interdependent acquisition parameters: the contrast agent dose and the image acquisition time [16]. Excessive microbubble concentrations may lead to signal overlap and localization errors, whereas insufficient doses prolong acquisition time and increase susceptibility to respiratory and cardiac motion artifacts [17, 18]. Importantly, current renal ULM studies often employ contrast doses empirically adapted from conventional CEUS protocols (*e.g*., 1.2–2.4 mL of sulfur hexafluoride SonoVue®, Bracco, Milan, Italy) [19, 20] without systematic validation for super-resolution imaging. Consequently, the absence of standardized, evidence-based acquisition protocols remains a major challenge to the reproducible clinical implementation of renal ULM.

Given the limited evidence regarding minimal effective dose and acquisition time for renal ULM in humans, this prospective study systematically evaluated their effects on cortical microvascular imaging in healthy volunteers. Secondary objectives included assessment of protocol feasibility, safety, and repeatability, as well as exploratory analysis of quantitative ULM-derived microvascular parameters.

## Materials and methods

### Subjects

This prospective study conformed to the Declaration of Helsinki and was reviewed and approved by the Institutional Review Board of The Second Affiliated Hospital of Nanjing Medical University (Approval No: 2024-KY-276-01). Healthy participants undergoing routine health examinations, with no known renal disease, were recruited from the hospital’s Health Examination Center between March and May 2025. All participants provided written informed consent.

The inclusion criteria were as follows: (1) aged 18–70 years; (2) normal renal morphology on ultrasound, as confirmed by the study sonographer, with assessment of size, cortical echogenicity, corticomedullary differentiation, and the absence of focal lesions; (3) no history of hypertension, hyperuricemia, diabetes or taking drugs causing kidney injury; (4) normal renal function was defined according to our institutional laboratory standards and clinical guidelines as: serum creatinine within 46–92 μmol/L; urea within 2.5–6.1 mmol/L; uric acid within 142.8–339.2 μmol/L; and estimated glomerular filtration rate–eGFR > 90 mL/min/1.73 m² (CKD-EPI equation [21]).

The exclusion criteria were: (1) contraindications for CEUS [22], *i.e*., hypersensitivity to sulfur hexafluoride or any component of SonoVue®, severe pulmonary hypertension, or right-to-left cardiac shunts; and (2) inability to comply with breath-holding instructions, which would result in significant motion artifacts.

### Sample size

The sample size was calculated a priori using G*Power software, version 3.1.9.7 (Heinrich-Heine-Universität Düsseldorf). The calculation was based on a repeated-measures ANOVA with one within-subjects factor (four contrast doses), assuming a medium effect size (f = 0.25) [23], an α error of 0.05, and a power of 80%. Under these assumptions, a minimum of 16 participants was required. Ultimately, 25 participants were enrolled and completed the study.

### Ultrasound image acquisition

The right kidney of each participant was examined using CEUS and ULM with four contrast doses (0.4, 0.6, 0.8, and 1.2 mL) to ensure standardized and consistent imaging acquisition. The injection order was randomized using a computer-generated sequence (Microsoft Excel version 2,607, Microsoft), ensuring equal probability across all 24 possible dose orders. Allocation was concealed from the sonographer until injection. All examinations were performed by a sonographer with 15 years of ultrasound and 5 years of CEUS experience.

### Preparation and positioning

The participants were positioned in the left lateral decubitus position to optimize acoustic access to the right kidney. Prior to image acquisition, all participants underwent standardized prescan training to ensure they could perform a stable 10-s breath-hold. Breath-holding was initiated at end-expiration during data acquisition to minimize respiratory motion and reduce tracking errors, thereby improving the stability and reliability of ULM measurements.

### Ultrasound system and imaging mode

All scans were performed using an ULTIMUS 9E system (VINNO Technology) with an S1-8C convex probe. Ultrafast plane-wave CEUS was conducted at frame rates up to 300 Hz with a low mechanical index of 0.059. Gain and depth were optimized prior to scanning, and the VSpeckle parameter was set to 4.

### Multidose contrast agent injection

The participants received four randomized intravenous bolus injections SonoVue® (Bracco) at 0.4, 0.6, 0.8, and 1.2 mL (estimated concentrations: 0.8, 1.2, 1.6, and 2.4 × 10⁸ microbubbles, respectively) *via* a 20-gauge antecubital cannula. Each injection was flushed with 5.0 mL of 0.9% saline, and the participants were closely monitored for adverse reactions. A 15-min washout interval was observed.

### Multiphase ULM data acquisition during CEUS

To characterize the temporal evolution of renal microvascular perfusion, a 120-s CEUS video clip was recorded immediately after contrast injection. This acquisition time was designed to encompass the major enhancement phases of the kidney, including the cortical phase (10–30 s), medullary phase (30–60 s), and delayed phase (> 60 s) [24].

For ULM analysis, datasets were retrospectively extracted from the CEUS recording at predefined time windows corresponding to these phases: 15–25 s (cortical phase, including the peak enhancement phase), 35–45 s (medullary phase), and 75–85 s (delayed phase). These time points were selected to represent distinct perfusion states while maintaining sufficient microbubble signal density for reliable localization and tracking. Consequently, each examination yielded one 120-s renal perfusion CEUS cine loop and three phase-specific ULM raw datasets for subsequent quantitative analysis.

### Image analysis

**Fig. 1:**
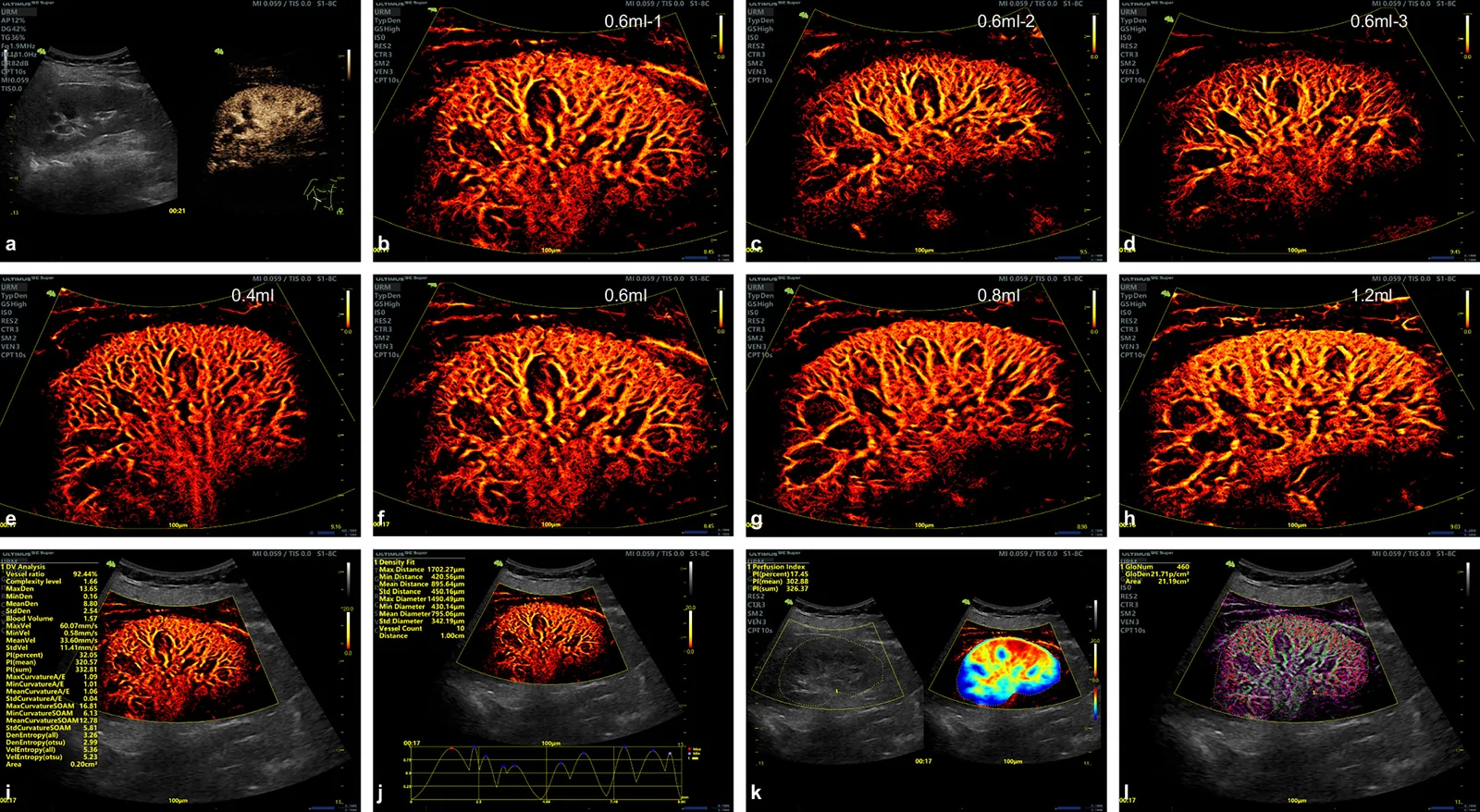
Representative images of ultrasound localization microscopy (ULM) and quantitative analytical parameters. **a** Contrast-enhanced ultrasound (CEUS) image; **b**–**d** ULM images acquired with a contrast agent dose of 0.6 mL at three time points: 15–25 s, 35–45 s, and 75–85 s; **e**–**h** ULM images acquired at four contrast doses (0.4, 0.6, 0.8, and 1.2 mL) during the 15–25 s phase; **i** microvascular density and velocity analysis; **j** vascular morphometry image; **k** perfusion parameters; **l** glomerular number.

The representative imaging features and quantitative analytical parameters for both CEUS and ULM are summarized in Fig. 1. This integrated analytical framework enabled a comprehensive assessment of renal cortical microvascular morphology, density, flow dynamics, and glomerular distribution.

#### CEUS

Quantitative perfusion analysis was performed using the system’s integrated software. To ensure representative sampling and reduce the influence of local perfusion heterogeneity, a standardized regions of interest (ROIs) placement protocol was implemented. Specifically, three non-overlapping circular ROI, each measuring 0.1 cm², were carefully positioned in the mid-renal cortex, approximately 1–2 mm beneath the renal capsule. ROI placement avoided large vessels to ensure consistency and comparability across participants and doses. Time–intensity curves were generated for each ROI, and perfusion parameters, including area under the curve, peak intensity, half-time to peak, time to peak, arrival time, and acceleration time, were extracted. The final quantitative values for each parameter were calculated as the mean of the three ROI measurements, providing a representative assessment of cortical microvascular perfusion.

#### ULM

ULM analysis was performed using integrated super-resolution software, version 1.51.36 (VINNO Technology). Motion correction algorithms were applied automatically, and frames affected by respiratory out-of-plane motion were excluded to minimize artifacts. Microvascular reconstruction followed a sub-100 μm localization workflow, which included microbubble detection, tracking, and trajectory mapping. All analyses were conducted by trained analysts following standardized operating procedures, using consistent software settings across all participants and imaging sessions to ensure reproducibility. The analyzed parameters encompassed multiple dimensions of cortical microvascular structure and function, including vascular density, flow velocity, perfusion, morphometric characteristics, and glomerular number.

#### Microvascular density and velocity analysis

Following microbubble detection, sub-pixel localization, and trajectory reconstruction (with velocity constraints and persistence filtering), super-resolution microvascular density and velocity maps were generated. For quantitative analysis, a 0.2 cm² ROI was placed in the subcapsular cortex (1–2 mm depth, mid-kidney). Each reported parameter represented the mean of three independent measurements. The following parameters were extracted from these maps within each ROI:

- vessel ratio = percentage of ROI area occupied by vasculature in the binarized density map;
- complexity level = fractal dimension of the vascular skeleton, reflecting morphological complexity;
- maximum density/mean density = peak and mean pixel intensity of the density map within the ROI, indicating microbubble signal abundance;
- maximum velocity/mean velocity = peak and mean values of instantaneous velocities from all tracked microbubbles within the ROI;
- density entropy/velocity entropy: Shannon entropy [25] of the spatial distribution of microbubble localizations and the velocity magnitude histogram, respectively, quantifying perfusion and flow heterogeneity.
- maximum curvature/mean curvature = maximum and mean values of vessel curvature from all tracked vessels within the ROI, reflecting the degree of local vessel bending.

#### Density fit (morphology)

A 1-cm sampling line perpendicular to the ultrasound beam was placed in the subcapsular cortex. The following parameters were derived using density-fit modeling and averaged across three measurements:

- mean vessel diameter = mean diameter of vessels intersecting the sampling line;
- mean intervascular distance = mean distance between adjacent vessels along the sampling line;
- vessel count = number of vessels intersecting the sampling line per centimeter.

#### Perfusion parameters

To assess global renal perfusion, the entire kidney contour was manually traced on ULM images. The perfusion index was then calculated as the product of mean flow velocity and vessel ratio within this global region using the formula: perfusion index = mean velocity × vessel ratio. For each participant, the final perfusion index value was calculated as the mean of three independent measurements. This composite parameter integrates microvascular flow dynamics and vascular density into a single quantitative metric, reflecting the overall perfusion status of the kidney.

#### Glomerular number

The principle of glomeruli counting in ULM is based on identifying microbubble tracks with exceptionally high tortuosity, quantified by a normalized distance metric. Tracks exhibiting a significantly longer path length relative to their linear displacement are classified as originating from the coiled glomerular capillaries. These high-distance metric tracks are then spatially clustered to localize and count individual glomeruli within the super-resolved microvascular map [26, 27]. Glomerular number was quantified from three manual segmentations of the entire renal cortex, with the final value representing the mean of these measurements to enable global assessment of glomerular distribution.

### Reproducibility analysis

To assess the reproducibility of CEUS and ULM parameter measurements, intra-observer and inter-observer reliability analyses were performed on a randomly selected subset of 10 participants. For intra-observer reliability, the same investigator (investigator A, the sonographer who performed all study scans with 15 years of experience in ultrasound and 5 years in CEUS) who conducted the primary ULM analysis repeated the entire workflow (including ROI placement and parameter extraction) after a 2-week interval, blinded to the previous measurements. For inter-observer reliability, a second independent observer (investigator B, a senior ultrasonographer with 10 years of experience in renal ultrasound, blinded to the results obtained by investigator A) independently performed the same analysis on the same 10 participants.

### Criteria for minimal effective contrast dose selection

The minimal effective contrast dose was predefined as the lowest dose at which all quantitative parameters that showed a significant dose–response relationship reached a statistical plateau. A plateau was defined as no significant pairwise difference between that dose and higher doses (0.8 mL or 1.2 mL) on *post hoc* testing with Bonferroni correction, while still being significantly superior to the lowest dose (0.4 mL).

### Statistical analysis

Statistical analysis was performed using IBM SPSS Statistics (version 27.0; IBM Corp.) and GraphPad Prism (version 10.0; GraphPad Software, LLC). Continuous variables were tested for normality and are presented as the mean ± standard deviation. Non-normally distributed data are expressed as medians with their interquartile ranges.

One-way repeated-measures ANOVA was used for CEUS parameters, and two-way repeated-measures ANOVA for ULM parameters (dose × time). Greenhouse–Geisser correction [28] was applied when sphericity was violated. *Post hoc* comparisons were performed with Bonferroni correction. Significance was set at *p* < 0.05 (two-sided).

The intra- and inter-observer reproducibility in measuring ultrasound parameters was evaluated by the intraclass correlation coefficient (ICC). The ICC values were interpreted according to the proposed standards: < 0.50 poor, 0.50–0.75 moderate, 0.75–0.90 good, and > 0.90 excellent [29].

## Results

### Baseline characteristics

**Fig. 2:**
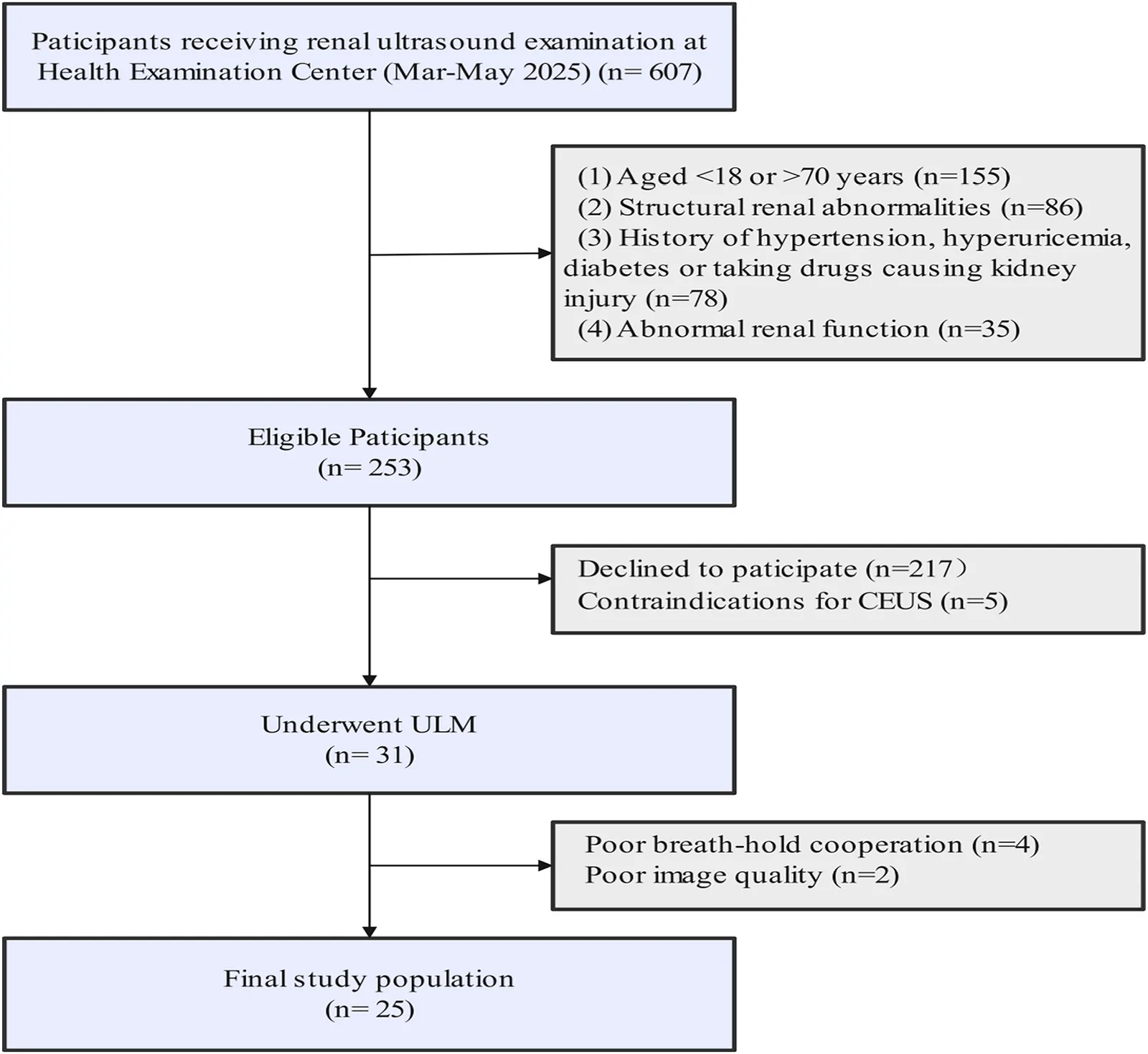
Flowchart of the study design

This prospective study enrolled 25 healthy participants (8 males and 17 females) with a median age of 29.0 years (range: 22–66 years), including 6 participants aged ≥ 50 years. Participant eligibility assessment and the recruitment flow are illustrated in Fig. 2. The baseline demographic and clinical characteristics of the cohort, including relevant laboratory and anthropometric parameters, are summarized in Table 1. Body mass index ranged from 16.8 to 29.8 kg/m². According to the World Health Organization international classification, 5 participants were underweight (< 18.5 kg/m²), 14 were normal weight (18.5–24.9 kg/m²), and 6 were overweight (25.0–29.9 kg/m²); no participants had obesity (≥ 30 kg/m²).

**Table 1:** Baseline characteristics of the study participants

Characteristics	*n* = 25
Demographics	
Age (years)	29.0 (25.5, 47.0)
Male/female	8/17 (32/68%)
Body mass index (kg/m^2^)	22.5 ± 4.0
Vital signs	
Heart rate (bpm)	75.9 ± 8.3
Systolic blood pressure (mmHg)	121.8 ± 6.5
Diastolic blood pressure (mmHg)	76.1 ± 5.6
Mean arterial pressure (mmHg)	91.3 ± 3.9
Renal function	
Serum creatinine (μmol/L)	68.2 ± 13.4
eGFR (mL/min/1.73 m²)	114.7 ± 9.8
Urea (mmol/L)	4.9 ± 1.1
Uric acid (μmol/L)	296.3 ± 63.4
Cardiac function	
Left ventricular ejection fraction (%)	65.6 ± 3.7

Data are presented as mean ± standard deviation except for age, as the variable was not normally distributed, thus presented as median (interquartile range, IQR). Left ventricular ejection fraction values (*n* = 20) derived from archived clinical echocardiographic data (≤ 12 months prior to the study visit); *n* = 5 measured by transthoracic echocardiography on the day of ultrasound localization microscopy examination

*eGFR* Estimated glomerular filtration rate

### Safety and exam completion

All twenty-five enrolled participants successfully completed the study imaging protocol, and all planned CEUS and ULM datasets were obtained for analysis. The sulfur hexafluoride microbubble contrast agent was well tolerated at all administered dose levels. No immediate or delayed adverse events were reported during or after the examination period, and no participants required premature termination of the procedure due to intolerance or safety concerns.

### CEUS parameters

**Fig. 3:**
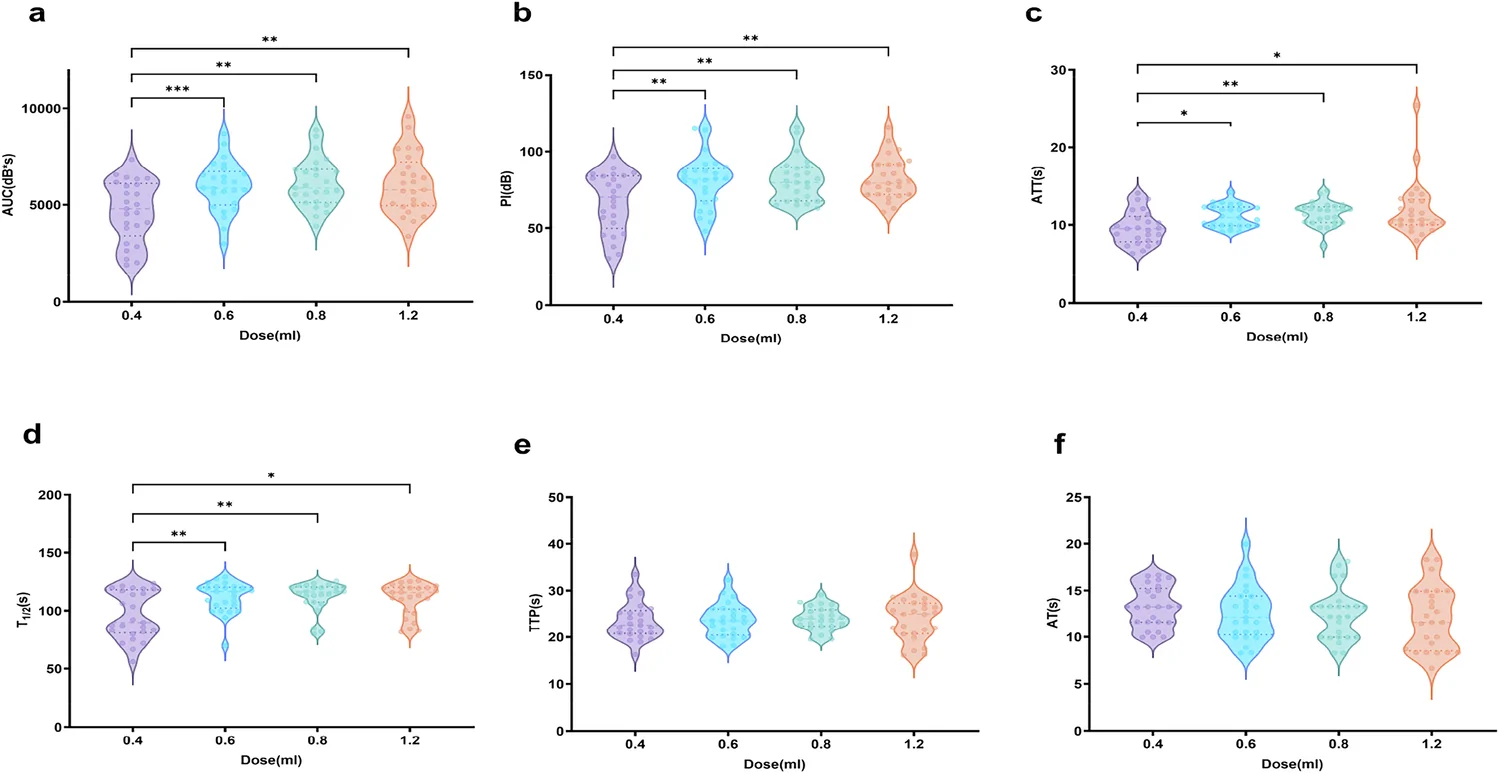
Comparison of contrast-enhanced ultrasound (CEUS) parameters by four contrast doses. Distribution of quantitative CEUS parameters (**a**–**f**) at four contrast doses (0.4, 0.6, 0.8, and 1.2 mL). Data are presented as violin plots with internal box plots (median, interquartile range (IQR)). Significant pairwise differences are indicated (* *p* < 0.05, ** *p* < 0.01, *** *p* < 0.001; repeated-measures ANOVA with Bonferroni correction)

CEUS parameters demonstrated a clear dose–response relationship, with a performance plateau emerging at doses ≥ 0.6 mL. One-way repeated-measures ANOVA showed significant overall effects of contrast dose on area under the curve, peak intensity, half-time to peak, and acceleration time (*p* ≤ 0.008; Table 2). *Post hoc* Bonferroni-corrected comparisons confirmed that the 0.4 mL dose yielded significantly lower values than each of the three higher doses (0.6, 0.8, and 1.2 mL) (*p* ≤ 0.031), while no significant differences were observed among the 0.6, 0.8, and 1.2 mL doses themselves, indicating that doses above 0.6 mL provided no additional enhancement benefit, further supporting that 0.6 mL represents the threshold dose for minimal effective CEUS enhancement. In contrast, arrival time and TTP showed no significant dose-dependent effects in either the overall ANOVA or *post hoc* analyses (Fig. 3 and Table 2).

**Table 2:** Effects of contrast dose on quantitative contrast-enhanced ultrasound (CEUS) parameters in healthy volunteers (*n* = 25)

Parameter	Dose 0.4 mL	Dose 0.6 mL	Dose 0.8 mL	Dose 1.2 mL	F(df1,df2)	*p*-value	*Post hoc* comparisons
Area under the time–intensity curve (dB·s)	4,676.4 ± 1,588.2	5,951.3 ± 1,309.9	6,112.1 ± 1,260.4	6,078.0 ± 1,576.5	11.278 (2.348, 56.35)	< 0.001	0.4 < 0.6, 0.8, 1.2
Peak intensity (dB)	66.8 ± 19.3	80.2 ± 16.9	81.2 ± 14.3	82.3 ± 13.9	9.277 (2.287, 54.89)	< 0.001	0.4 < 0.6, 0.8, 1.2
Half-time to peak (s)	95.6 ± 20.5	111.4 ± 13.2	112.2 ± 12.8	110.2 ± 14.1	9.251 (2.678, 64.26)	< 0.001	0.4 < 0.6, 0.8, 1.2
Acceleration time (s)	9.6 ± 2.1	11.1 ± 1.4	11.4 ± 1.5	12.0 ± 3.6	5.189 (2.082, 49.96)	0.008	0.4 < 0.6, 0.8, 1.2
Arrive time (s)	13.2 ± 2.2	12.5 ± 2.9	12.4 ± 2.7	12.0 ± 3.4	1.891 (2.548, 61.14)	0.149	NS
Time to peak (s)	23.5 ± 4.0	23.7 ± 3.6	24.0 ± 2.5	24.1 ± 4.9	0.160 (2.334, 56.01)	0.880	NS

Data are presented as mean ± standard deviation. *Post hoc* comparisons: *p* < 0.05 *versus* 0.4-mL dose (Bonferroni-corrected)

*df* Degrees of freedom, Greenhouse–Geisser corrected, *F* Test statistic for repeated-measures ANOVA, *NS* Not significant

### ULM parameters

Stratified analysis of ULM parameters showed distinct patterns of different contrast doses and acquisition times. Two-way repeated-measures ANOVA was performed to investigate the individual and interactive effects of contrast dose and time phase on ULM parameters. No significant interaction effects were observed for any parameter (*p* ≥ 0.062). On the basis of their distinct response patterns to the main effects, the parameters were stratified into four groups with complete statistical details provided in Table 3.

**Table 3:** Effects of contrast dose and time on ultrasound localization microscopy (ULM) parameters (*n* = 25)

Parameter category and name	Dose effect F(df1, df2)	*p*-value	Time effect F(df1, df2)	*p*-value	*Post hoc* pattern
Dual-influenced (dose and time)					
Vessel ratio (%)	F(3, 96) = 4.097	0.009	F(1.515, 145.4) = 112.3	< 0.001	0.4 < 0.6, 0.8, 1.2
Maximum velocity (mm/s)	F(3, 96) = 4.152	0.008	F(1.977, 189.8) = 62.11	< 0.001	0.4 < 0.6, 0.8, 1.2
Mean velocity (mm/s)	F(3, 96) = 5.071	0.003	F(1.599, 153.5) = 219.2	< 0.001	0.4 < 0.6, 0.8, 1.2
Dose-dependent only					
Vessel count	F(3, 96) = 2.805	0.044	F(1.932, 185.5) = 0.013	0.986	0.4 < 0.6, 0.8, 1.2
Time-dependent only					
Complexity level	F(3, 96) = 0.920	0.435	F(1.365, 131.0) = 39.77	< 0.001	-
Maximum density	F(3, 96) = 0.805	0.494	F(1.912, 183.6) = 19.50	< 0.001	-
Mean density	F(3, 96) = 2.565	0.059	F(1.851, 177.7) = 48.10	< 0.001	-
Velocity entropy (all)	F(3, 96) = 0.520	0.670	F(1.626, 156.1) = 11.62	< 0.001	-
Mean diameter (μm)	F(3, 96) = 0.304	0.822	F(1.757, 168.7) = 89.24	< 0.001	-
Perfusion index	F(3, 96) = 1.620	0.190	F(1.544, 148.3) = 18.93	< 0.001	-
Glomerular number	F(3, 96) = 0.330	0.803	F(1.961, 188.2) = 13.66	< 0.001	-
Stable parameters					
Maximum curvature	F(3, 96) = 0.323	0.809	F(1.816, 174.3) = 2.829	0.067	-
Mean curvature	F(3, 96) = 1.616	0.191	F(1.722, 165.3) = 3.071	0.057	-
Density entropy (all)	F(3, 96) = 0.312	0.817	F(1.894, 181.8) = 1.619	0.531	-
Mean distance (μm)	F(3, 96) = 1.723	0.168	F(1.639, 162.5) = 0.970	0.369	-

*Post hoc* tests (with Bonferroni correction) showed significantly lower values at the 0.4 mL dose than at the 0.6, 0.8, and 1.2 mL doses (*p* < 0.05). No significant differences were observed among 0.6, 0.8, and 1.2 mL doses

#### Parameters affected by both contrast doses and time

**Fig. 4:**
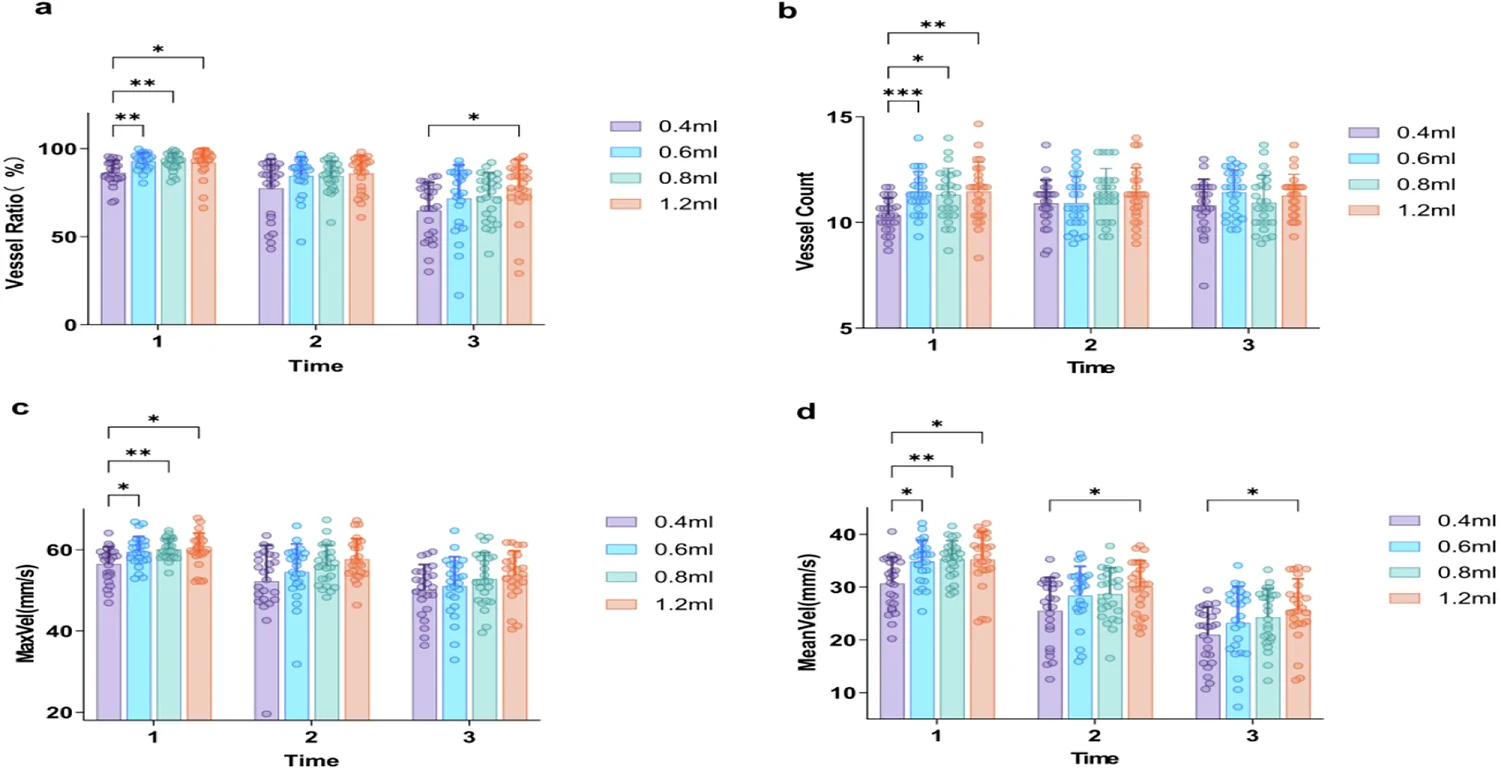
Dose-dependent effects on hemodynamic parameters. Bar graphs show the values of (**a**) vessel ratio, (**b**) vessel count, (**c**) maximum velocity (MaxVel), and (**d**) Mean velocity (MeanVel) across four contrast doses at three different phases. Data are presented as mean ± standard deviation (*n* = 25). Asterisks indicate a significant main effect of dose following Bonferroni correction (*p* < 0.05 compared to the 0.4 mL group)

A group of parameters, comprising the vessel ratio, max velocity, and mean velocity, was sensitive to both experimental factors (Fig. 4). These parameters showed a significant main effect of contrast dose (*p* ≤ 0.009) as well as a significant main effect of time (all *p* < 0.001). This dual sensitivity indicates that these parameters are the most responsive to imaging conditions and are therefore key candidates for protocol optimization.

#### Parameters affected exclusively by contrast dose

The vessel count was affected by the contrast doses, demonstrating a significant main effect for this factor (*p* = 0.044) but not for time (*p* ≥ 0.986). This suggests that vessel count is primarily determined by contrast agent availability rather than perfusion phase, making it a robust parameter across different acquisition times.

#### Parameters affected exclusively by time

A group of parameters, including complexity level, maximum density, mean density, velocity entropy, mean diameter, perfusion index, and glomerular number, exhibited significant changes over time (*p* < 0.001) but were unaffected by contrast dose (*p* ≥ 0.059). These time-dependent parameters likely reflect physiological variations in renal perfusion across different phases, and their dose-independence suggests they can be reliably compared across studies using different contrast doses.

#### Parameters unaffected by contrast doses and time

The parameters maxicurvature, mean curvature, density entropy, and mean distance remained stable across contrast doses and time (*p* ≥ 0.057). This stability suggests that these morphometric parameters are relatively insensitive to dose and timing variations, supporting their role as stable structural descriptors of the renal microvasculature.

### Reproducibility of CEUS and ULM parameters

**Fig. 5:**
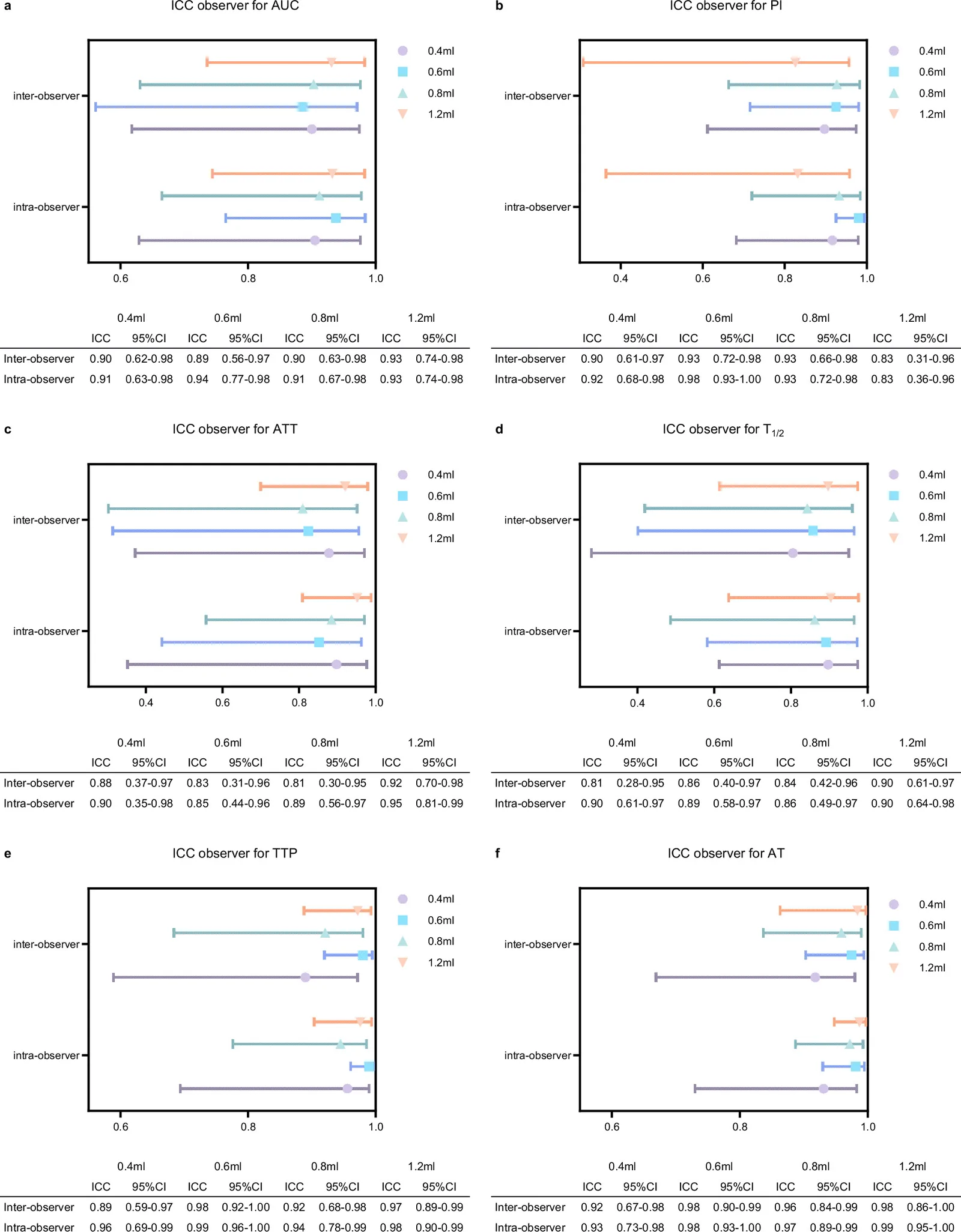
The inter- and intra-observer intraclass correlation coefficients (ICCs) and 95% confidence intervals (CIs) of measurement for four doses of contrast-enhanced ultrasound (CEUS) parameters: (**a**) measurements of area under the time–intensity curve (AUC); (**b**) measurements of peak intensity (PI); (**c**) measurements of acceleration time (ATT); (**d**) measurements of half-time to peak (T1/2); (**e**) measurements of time to peak (TTP); and (**f**) measurements of arrive time (AT)

**Fig. 6:**
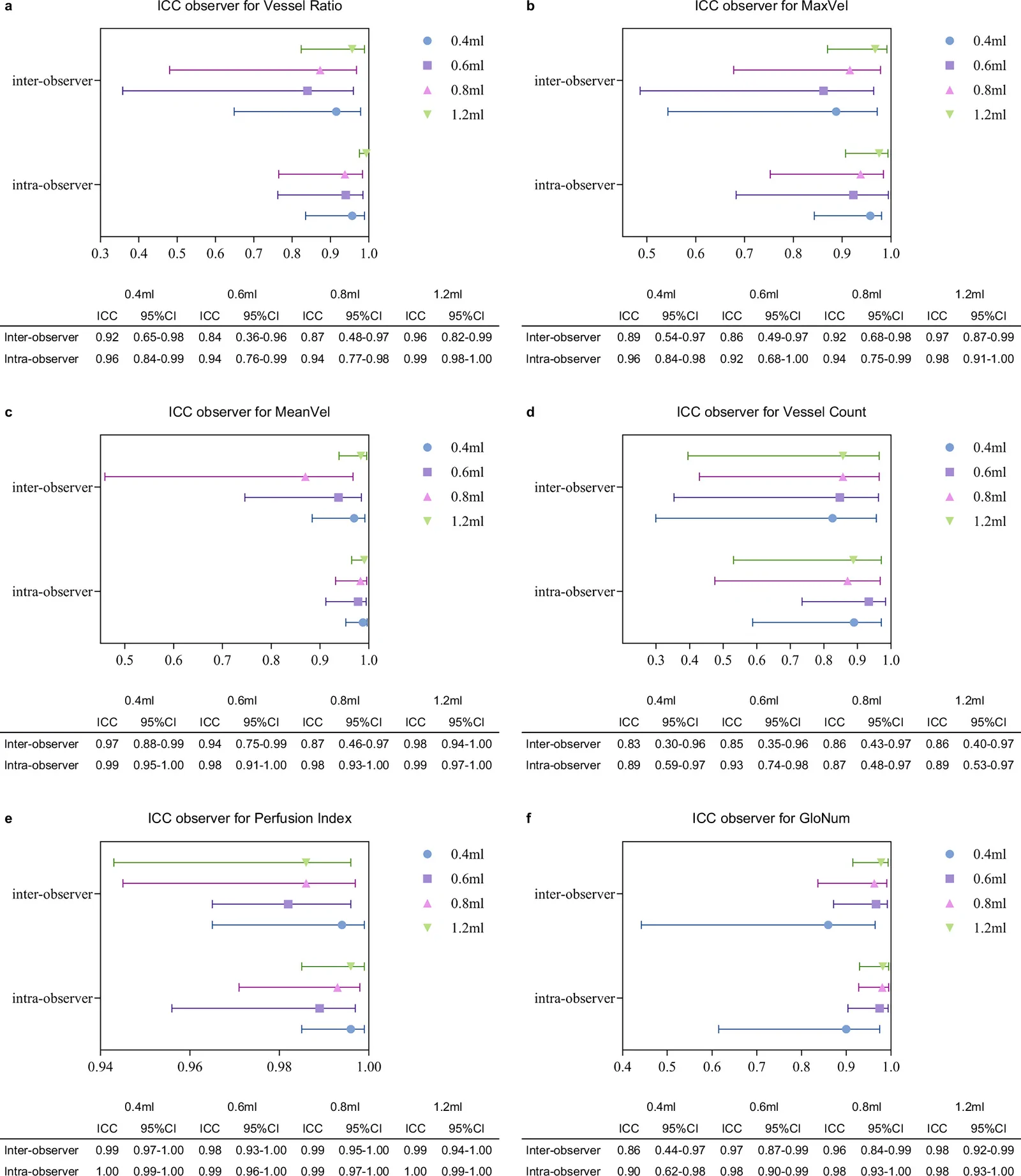
Inter- and intra-observer intraclass correlation coefficients (ICCs) and 95% confidence intervals (CIs) of measurement for four doses of ultrasound localization microscopy (ULM) parameters: (**a**) measurements of vessel ratio; (**b**) measurements of maximum velocity (MaxVel); (**c**) measurements of mean velocity (MeanVel); (**d**) measurements of vessel count; (**e**) measurements of perfusion index; and (**f**) measurements of glomerular number (GloNum)

Both CEUS and ULM parameters demonstrated good to excellent reproducibility across all contrast doses. For CEUS parameters, intra-observer ICCs ranged from 0.83 to 0.99, and inter-observer ICCs ranged from 0.81 to 0.98 across the four administered doses. For ULM parameters, intra-observer ICCs ranged from 0.87 to 1.00, and inter-observer ICCs ranged from 0.83 to 0.99. According to established ICC interpretation criteria, all parameters demonstrated good to excellent reproducibility. Detailed ICC values with corresponding 95% confidence intervals for each parameter at each dose level are presented in Figs. 5 and 6.

## Discussion

This exploratory study evaluated the effects of contrast dose and acquisition time on renal cortical microvascular imaging using ULM in healthy participants. Our findings suggest that 0.6 mL of sulfur hexafluoride microbubbles may represent a minimal effective dose, as both CEUS (area under the curve, peak intensity, half-time to peak, acceleration time) and core ULM parameters (vessel ratio, maximum velocity, mean velocity) reached a performance plateau comparable to higher doses. The 0.4 mL dose did not reach this plateau, while doses above 0.6 mL increased contrast exposure without additional imaging benefit. Optimization of acquisition time was another critical parameter, with the peak enhancement phase providing a period of maximal and relatively homogeneous microbubble distribution that improved signal stability and parameter reproducibility.

The stratified response patterns observed in this study indicate that renal ULM parameters differ in their sensitivity to contrast dose and acquisition time. Flow-related metrics (vessel ratio, maximum velocity, mean velocity) were influenced by both factors, underscoring the need for strict protocol standardization when used for quantitative comparisons. Vessel count was influenced exclusively by contrast dose, suggesting it primarily reflects vascular density and remains robust across different acquisition phases. Perfusion-related indices (complexity level, maximum density, mean density, velocity entropy, mean diameter, perfusion index, glomerular number) varied across phases but remained dose-independent, indicating greater robustness for longitudinal or comparative applications. Morphometric descriptors (maximum curvature, mean curvature, density entropy, mean distance) remained stable across all conditions, supporting their role as structural rather than dynamic markers. This differential sensitivity may assist in rational parameter selection according to specific research objectives and study designs.

In our study, the need for a low contrast dose observed is consistent with the physical principles established by Lerendegui et al [18], who demonstrated that significant localization errors occur when inter-microbubble spacing falls below 1.7 to 3.0 times the point spread function; at higher concentrations, adjacent microbubble signals overlap, leading to tracking ambiguity and reduced localization precision. In contrast, the initial in-human feasibility study by Huang et al employed a standard clinical dose [7], a practical choice that prioritized reliable signal detection rather than optimization of localization accuracy. This distinction suggests a shift in emphasis from feasibility toward protocol optimization for quantitative assessment.

In this context, our findings provide initial human data supporting protocol refinement toward quantitative accuracy. While prior research established the theoretical basis for low-dose imaging and demonstrated in-human feasibility using clinical doses, our study extends these concepts by systematically evaluating dose and acquisition timing within a renal-specific framework. This dose is lower than conventional renal CEUS doses (1.2–2.4 mL) [19], potentially reducing contrast exposure for repeated examinations. Within the tested range, 0.6 mL achieved quantitative performance comparable to higher doses, indicating that a reduced-dose protocol is feasible without compromising ULM parameters. Notably, these dosing requirements may be organ-specific; the kidney’s high cortical perfusion and respiratory motion increase the risk of signal overlap. Thus, the minimal effective dose for renal ULM may not directly generalize to other vascular territories without dedicated validation.

In terms of parameter analysis, ULM, as an emerging technology, offers distinct advantages over conventional CEUS for microvascular imaging, while also facing significant challenges. ULM overcomes the diffraction limit of ultrasound, enabling unprecedented visualization of *in vivo* microvascular structures in detail. This allows for objective and quantitative analysis of vascular networks in terms of velocity, tortuosity, and distribution [6, 11]. More importantly, ULM provides operator-independent absolute hemodynamic parameters such as relative blood volume and blood flow velocity, achieving direct and quantitative assessment of microvascular function [3, 30]. This fundamentally differs from the indirect and semi-quantitative evaluation of CEUS based on time–intensity curves. Conventional Doppler ultrasound, while widely accessible, remains constrained by the acoustic diffraction limit and generally provides semi-quantitative or macrovascular perfusion information rather than direct microvascular mapping.

These advantages extend beyond ultrasound-based techniques when compared with other imaging modalities. Dynamic contrast-enhanced MRI and CT can provide indirect perfusion assessment through contrast kinetics; however, their spatial resolution (typically > 500 μm) remains insufficient to resolve cortical microvessels [31]. In addition, concerns regarding gadolinium exposure or ionizing radiation may limit repeated examinations, particularly in patients with impaired renal function. Micro-CT, although capable of high-resolution vascular visualization, is restricted to *ex vivo* or experimental settings and does not allow dynamic *in vivo* assessment [4]. Despite these advantages, ULM’s limitations are equally prominent: currently, ULM imaging is more time-consuming (including both data acquisition and post-processing), technically more complex, and often requires dedicated research support. Most critically, while clinical ultrasound and CEUS are real-time imaging techniques, ULM, due to its lengthy acquisition and processing times, is not yet available for real-time imaging. This restricts its immediate application in clinical scenarios requiring dynamic observation of perfusion processes [6].

Despite these findings, several limitations should be acknowledged. First, this was a single-center study performed using a single ultrasound platform and dedicated software, which may limit the generalizability and external validity of the findings. The study cohort consisted of a relatively small number of healthy participants with a body mass index range of 16.8–29.8 kg/m², covering underweight, normal, and overweight categories, but did not include individuals with obesity (body mass index ≥ 30 kg/m²). Therefore, the optimized protocol is recommended for non-obese healthy adults, and validation in obese populations is needed. Second, the proposed 0.6 mL dose was derived from observations in normal kidneys and has not been validated in pathological conditions. Renal diseases may alter microvascular architecture and perfusion dynamics, potentially influencing contrast dose and acquisition time. Third, the timing of left ventricular ejection fraction assessment represents an additional limitation. Some left ventricular ejection fraction values were retrieved from clinical echocardiograms acquired up to 12 months before study participation. While these participants reported no cardiac symptoms or major cardiovascular events in this period, subtle temporal changes in left ventricular systolic function cannot be ruled out. In addition, only one sulfur hexafluoride microbubble contrast agent was evaluated, and the applicability of this protocol to other commercially available agents remains to be determined. Furthermore, ULM currently lacks an established *in vivo* reference standard for direct validation of reconstructed microvascular structures. Although the glomerular number provides a quantitative index derived from localization data, it should be regarded as an exploratory functional parameter rather than a direct anatomical count.

Future investigations should include validation against histopathological or established imaging references, application of the optimized protocol in patient cohorts, and multicenter assessments across different ultrasound platforms, software environments, and contrast agents to enhance reproducibility and clinical translatability.

## Conclusion

In conclusion, this study proposes a standardized renal ULM imaging protocol for healthy, non-obese adults (body mass index < 30 kg/m^2^). A 0.6-mL contrast dose with analysis during the peak enhancement phase enabled renal reproducible microvascular visualization. These findings provide a methodological reference for future studies investigating renal microvascular alterations.

## Data Availability

The datasets generated and/or analyzed during the current study are not publicly available but may be available from the corresponding author upon reasonable request.

## Abbreviations

CEUS: Contrast-enhanced ultrasound
ICC: Intraclass correlation coefficient
ROI: Region of interest
ULM: Ultrasound localization microscopy
